# A cost-benefit analysis of mass prostate cancer screening

**DOI:** 10.1186/s12962-024-00553-0

**Published:** 2024-05-05

**Authors:** Hiro Farabi, Najmeh Moradi, Aziz Ahmadzadeh, Seyed Mohammad Kazem Aghamir, Abdolreza Mohammadi, Aziz Rezapour

**Affiliations:** 1 Barts and the London Pragmatic Clinical Trials Unit, Centre for Evaluation and Methods, Wolfson Institute of Population Health, Queen Mary University of London, London, UK; 2 Population Health Sciences Institute, Newcastle University, Newcastle upon Tyne, UK; 3 Insurance Research Center (IRC), Tehran, Iran; 4 Urology Research Center, Tehran University of Medical Sciences, Tehran, Iran; 5 Health Management and Economics Research Center, School of Health Management and Information Sciences, Iran University of Medical Sciences, Tehran, Iran

**Keywords:** Cost-benefit analysis, Prostate cancer, Screening, Willingness to pay, Decision model, Health decision-making.

## Abstract

**Background:**

Prostate cancer (PCa) causes a substantial health and financial burden worldwide, underscoring the need for efficient mass screening approaches. This study attempts to evaluate the Net Cost-Benefit Index (NCBI) of PCa screening in Iran to offer insights for informed decision-making and resource allocation.

**Method:**

The Net Cost-Benefit Index (NCBI) was calculated for four age groups (40 years and above) using a decision-analysis model. Two screening strategies, prostate-specific antigen (PSA) solely and PSA with Digital Rectal Examination (DRE), were evaluated from the health system perspective. A retrospective assessment of 1402 prostate cancer (PCa) patients’ profiles were conducted, and direct medical and non-medical costs were calculated based on the 2021 official tariff rates, patient records, and interviews. The monetary value of mass screening was determined through Willingness to Pay (WTP) assessments, which served as a measure for the benefit aspect.

**Result:**

The combined PSA and DRE strategy of screening is cost-effective, yields up to $3 saving in costs per case and emerges as the dominant strategy over PSA alone. Screening for men aged 70 and above does not meet economic justification, indicated by a negative Net Cost-Benefit Index (NCBI). The 40–49 age group exhibits the highest net benefit, $13.81 based on basic information and $13.54 based on comprehensive information. Sensitivity analysis strongly supports the cost-effectiveness of the combined screening approach.

**Conclusion:**

This study advocates prostate cancer screening with PSA and DRE, is economically justified for men aged 40–69. The results of the study recommend that policymakers prioritize resource allocation for PCa screening programs based on age and budget constraints. Men’s willingness to pay, especially for the 40–49 age group which had the highest net benefit, leverages their financial participation in screening services. Additionally, screening services for other age groups, such as 50–54 or 55–59, can be provided either for free or at a reduced cost.

## Introduction

Cancer is one of the leading causes of death in the world. In the year 2020, Global forecasts expected a 45% increase in cancer in developing countries [1]. Cancer has a significant effect on the financial situation of patients and their families. It is estimated that 16.1 million people are living with cancer, faceing out-of-pocket payments 61% higher than patients with no history of cancer ($ 1,000 vs. $ 622). Cancer also has significant indirect costs in terms of lost revenue. An analysis of 493,000 deaths caused by cancer in people aged 16 to 84 in the United States in 2015 showed that $ 94.4 billion was lost in revenue [2].

Over the past decade, prostate cancer (PCa) has experienced rapid growth in Iran, emerging as one of the most prevalent types of cancers [3]. PCa imposes a substantial financial burden on healthcare systems worldwide. Treatment and hospitalization costs for PCa patients in European countries range between 106.7 and 179 million US dollars annually, while in the United States, they soar to 9.862 billion dollars. In Canada, the annual, inclusive of hospitalization and treatment, stands at 103.1 million dollars in 1998, whereas in Australia it was 101.1 million dollars between 1993 and 1994 [4]. In 2009, healthcare costs associated with prostate cancer across the EU totaled €8.43 billion [5]. The societal costs attributed to prostate cancer in Stockholm in 2016 were estimated at €64 million Euros, 62% of which related to direct medical costs [6].

Early detection of cancer not only yields positive clinical outcomes by reducing patient mortality rates and enhancing their quality of life, but also positively impacts treatment modalities and their financial implications. Numerous studies have demonstrated that early diagnosis leads to reduced treatment expenses. The cost of caring for cancer patients in their final years is significantly higher than caring costs during the initial phase following diagnosis [4] or the expenses associated with early-stage treatment [7, 8]. An economic burden study conducted in Iran found that patients with advanced-stage prostate cancer incur substantial treatment costs, whereas these expenses are two to three times lower in the early stages of treatment [3]. This discrepancy led to annual savings of $26 billion for early cancer detection in the United States [9]. Furthermore, early diagnosis enables patients to maintain their occupational activities by reducing treatment durations and incurring lower treatment costs [10].

Despite increasing prevalence and mortality rates of PCa in some developing countries, evidence shows that mortality rates in some high-income countries have declined as a result of early detection. This early detection is attributed to the common use of different early detection methods in these countries [11], clearly demonstrating the importance of early diagnostic measures, including screening [12].

Previous studies have shown that PCa screening causes a significant (about 21%) reduction in the mortality rate of PCa patients in Australia [13] in Iran [12, 14] and even in Europe, despite the high prevalence rate [15].

Prostate cancer (PCa) in Iran has imposed a significant financial burden, which totaled around $25.8 million in 2019 [16]. This disease has had an increasing incidence rate in this country. PCa incidence increases with age, and it is very common from the age of 50. Therefore, as the Iranian baby boom of the 1980s has reached their 40s, the aging of the population will accelerate and it is expected that PCa incidence will have an increasing trend in Iran. Consequently, the combination of the high burden of this disease and the resulting high economic burden, along with the increasing aging of the population, shows the importance of providing a screening program.

In order to make decisions about the implementation of screening programs, it is necessary to consider the evidence-based economic evaluation of implementing this program in terms of costs and benefits [17]. This is the main objective of this study.

Using WTP is a common method to measure the benefit alongside detecting the acceptance of strategies in economic evaluation and cost benefit analysis of implementing cancer interventions [17–19]. Also, the willingness to pay approach has the capacity to compute false positive and negative results of diagnostic tests, which help promote the accuracy of evaluation indicators and the elimination of potential biases [20]. Based on these propositions, in this study the willingness to pay approach has been used as a benefit index to estimate the NCBI of PCa screening for different age groups.

## Method and design

The foundations of calculating the Net cost-benefit index (NCBI) of prostate cancer (PCa) screening in Iranian men aged 40 and above relied on costs and benefits information. We retrospectively explored the SEPAS Iranian Health Ministry database to extract and compute costs, utilizing a decision tree within a one-year time horizon. Additionally, the monetary value of screening for prostate cancer was extracted from an earlier study conducted by the authors which used the Willingness to pay method.

The analysis was carried out from the health system perspective, and all costs were reported in United State Dollars (1$ = 230,000 IRR, based on the market exchange rate) [21]. To account for the Iranian inflation rate and the banking system interest rate (serving as the risk-free rate) over the last 40 years, a discount rate of 20% was applied to intertemporal calculations.

### Population

We identified all patients specifically seeking services in hospitals during 2021 and undergoing treatments with action codes related to prostate cancer (PCa) to form our study sample. This group comprised 1402 patients, and their recorded data were extracted from the Iranian Health System Database (SEPAS).

To estimate the Iranian population requiring screening for PCa in 2021, we extrapolated from pertinent population data derived from the 2016 Iran population and housing census [22]. This extrapolation considered the population growth rate between the two most recent census years conducted in Iran (2011 and 2016).

### Model structure

**Fig. 1:**
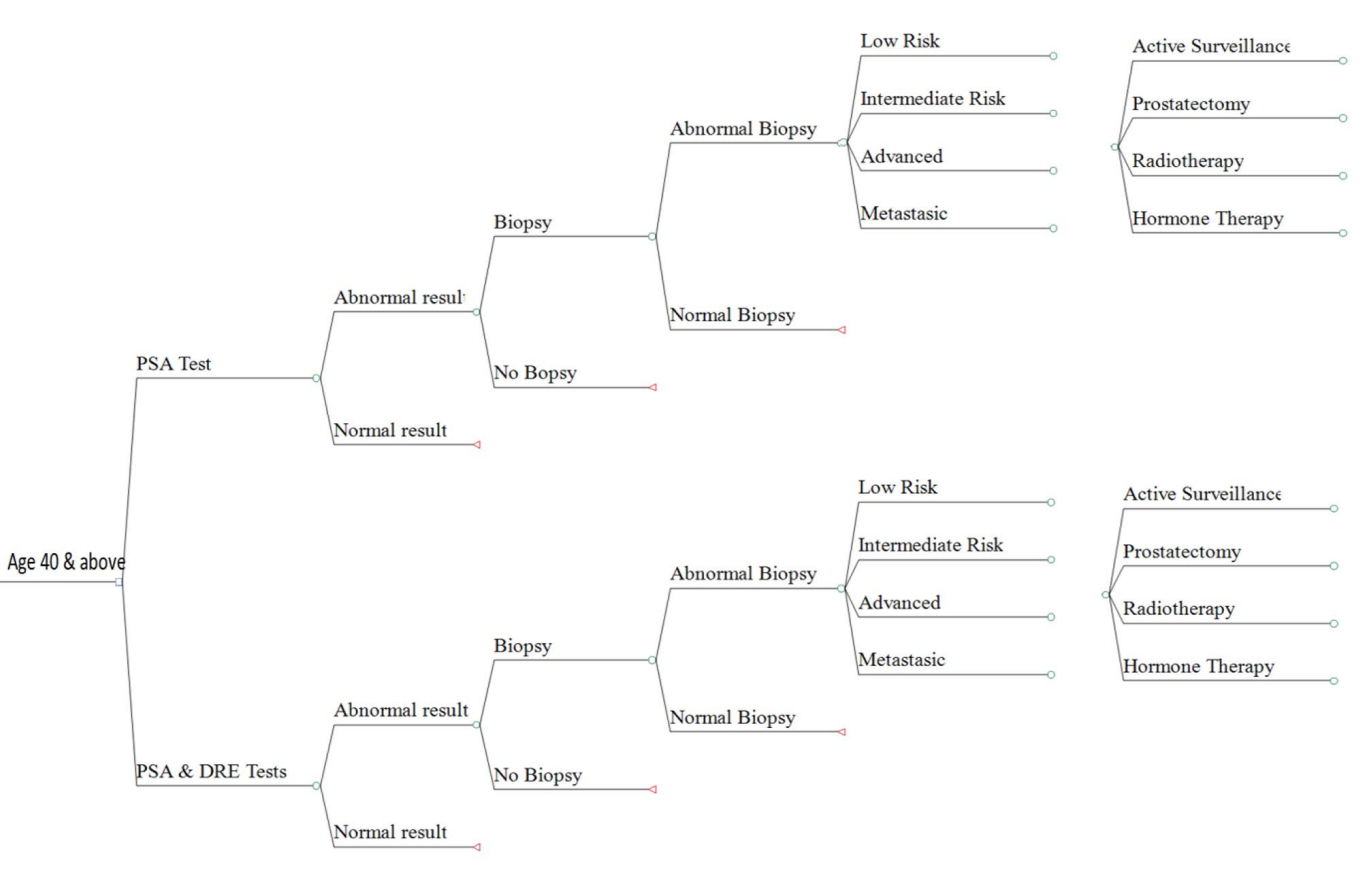
Prostate cancer screening decision tree

The model structure for our prostate cancer screening study compares two diagnostic tests: screening with a PSA test and screening with a combination of PSA and DRE tests. The decision tree (Fig. 1) and the associated probabilities (Table 1) were developed through a meticulous review of existing studies.

An expert panel, including four health economists and twelve urologists, customized the model to the context of this study, adjusting probabilities based on PSA cut-offs and different age groups. This tailored approach ensures the accuracy and relevance of our findings.

**Table 1:** Probabilities for entering next stage of screening in each node of the Decision Tree

Nodes	Criteria		Probability (%)	Treatment plan	Probability (%)
PSA test [8]	PSA > 2.5 ng/ml	(40–49) Age	2.4		
	PSA > 4.5 ng/ml	(50–54) Age	5.3		
	PSA > 6 ng/ml	(55–69) Age	2.6		
	PSA > 8 ng/ml	(70–79) Age	6.6		
	PSA > 10 ng/ml	80 +	10.3		
Doing Biopsy [23]	Any DRE		40.9–60.1		
	abnormal DRE		25		
			60.1		
			77		
Positive biopsy Probability [23]	Any DRE		44		
			56.1		
			60.1		
	abnormal DRE		34.1		
			63.7		
Stage of Disease [24]	Low Risk		36.3	Surveillance	9.2
				Prostatectomy	56.7
				Radiotherapy	26.4
				Hormone Therapy	7.6
	Intermediate Risk		36.3	Surveillance	4.8
				Prostatectomy	52.9
				Radiotherapy	30.3
				Hormone Therapy	11.9
	High Risk		15.1	Surveillance	3.2
				Prostatectomy	32.2
				Radiotherapy	31.7
				Hormone Therapy	32.8
	Unknown		12.4	Surveillance	9.9
				Prostatectomy	42.2
				Radiotherapy	28.9
				Hormone Therapy	18.9
Biopsy Complications [25]	Hematospermia		36.3		
	Hematuria		14		
	Bleeding lasting two days		2.3		
	Fever		0.8		
	Bleeding requires surgery		0.6		
	Urinary retention		0.2		
Prostatectomy Complication [26]			17		
Radiotherapy Complication [26]			1.6		

### Resource use and unit costs

In this study, we considered both direct medical and non-medical costs when calculating the prostate cancer screening cost.

Direct Medical Costs:

Direct medical costs included resource utilization for diagnosis, treatment, and associated complications.

The treatment costs for different prostate cancer (PCa) stages—low-, moderate-, and advanced-risk — included expenses related to prostatectomy surgery, radiotherapy, hormone therapy, and active surveillance regimens.

The complications costs, including bleeding, infection, sexual problems, urinary retention, cardiovascular issues, hot flashes, weakness, and lethargy, were comprehensively addressed in our study.

For inpatient resource utilization, we used action codes from the RVU book, extracting costs based on action codes from the SEPAS Iranian Health Ministry database and national tariffs.

The diagnostic costs were thoroughly considered for PSA, DRE, and biopsy tests, with the related resource use extracted from patient profiles and their unit costs derived from national tariffs.

Direct Non-Medical Costs:

The direct non-medical costs of PCa treatment were sourced from a relevant study estimating the economic burden of PCa in Iran.

The direct non-medical costs of PCa diagnosis were measured through phone interviews with a stochastic sample of 240 patients, covering transportation, travel, accommodation, and food expenses.

The total cost was defined as the combined sum of direct medical and non-medical costs. In each branch of our decision tree, average costs were computed by multiplying the costs associated with each node by their respective probabilities.

### Benefit

The assessment of benefits for prostate cancer screening in the current study builds upon insights gained from our prior research [27]. In this study, we utilized the willingness-to-pay method, incorporating two distinct sets of information. Participants’ monetary valuations were elicited through scenarios that presented comprehensive details, including both basic and complementary information related to prostate cancer diagnosis and treatment methods.

By employing this nuanced approach, we aimed to capture a more comprehensive understanding of individuals’ willingness to invest in prostate cancer screening. The combination of fundamental knowledge and supplementary insights provided participants with a holistic perspective, allowing for a more informed assessment of the perceived benefits associated with early detection.

This refined methodology ensures that our evaluation not only delves into the economic aspect of willingness to pay but also considers the impact of comprehensive information on participants’ decision-making processes. As a result, this study contributes a more nuanced and insightful perspective to the assessment of the benefits associated with prostate cancer screening.

### Net cost-benefit index

The Net Cost-Benefit Index (NCBI) was computed for all population and five distinct age groups of men (40–49, 50–54, 55–69, 70–79, and 80 & above) using the following formula:

***NCBI = Benefit*** *(Average of men’s willingness to pay)* ***– Cost*** *(Average cost of PCa screening)*

NCBI for prostate cancer (PCa) screening was determined under four conditions, encompassing two strategies for cost and two scenarios for benefit.

In interpreting the Net Cost-Benefit Index (NCBI), a positive value indicates that the average willingness-to-pay for prostate cancer (PCa) screening exceeds the average cost, suggesting a net benefit. Conversely, a negative value implies that the average cost outweighs the average willingness-to-pay. The calculation was performed under four conditions, considering different cost strategies and benefit scenarios, providing a comprehensive perspective on the economic implications of PCa screening across various age groups.

### Sensitivity analysis

A comprehensive one-way sensitivity analysis was undertaken, systematically varying the potential complications associated with both the screening and treatment processes by ± 20%. This rigorous exploration aimed to evaluate the impact of uncertainties surrounding complication rates on the findings of the present study. Subsequently, the robustness and reliability of the results were examined through an in-depth assessment of the Net Cost-Benefit Index (NCBI).

## Results

The population of Iranian men aged 40 years and older in 2021 are shown in Table 2 below.

**Table 2:** The population of Iranian men by Age in 2021

Age	*n*
Age (40–49)	4,667,517
Age (50–54)	1,864,652
Age (55–69)	2,746,324
Age (70–79)	1,035,339
Age 80+	370,143
Total	10,683,976

### Costs of prostate cancer screening

The results of the cost calculation with two different strategies indicate that the total cost of screening using PSA is approximately $127 million, while the cost of screening using both PSA and DRE equals approximately $90 million. Consequently, the cost of implementing this program with the combined DRE and PSA test is approximately 30% less as compared with conducting it with the PSA test alone (Table 3).

**Table 3:** Up to 40 men’s population prostate cancer screening costs with two strategies (USD)

Subject	Amount ($) All Men	
	Screening by PSA	Screening by PSA and DRE
**Screening Medical Cost**		
PSA Test	30,193,845	33,573,915
Biopsy & complication	15,088,842	3,370,128
Treatment & complication	37,173,353	11,767,247
Total Direct Medical Cost	82,456,040	48,711,289
**Screening non-Medical Cost**		
Direct Non-Medical Treatment Costs	12,623,136	13,144,348
Direct Non-Medical Diagnosis Costs	9,404,005	9,170,034
Total Direct Non-Medical Costs	22,027,140	22,314,382
**Total Costs**	104,483,181	71,025,671
Average Cost of Screening per man	10	7

The average cost of screening only through PSA was $10 per man, and it reduced to $7 per man with the inclusion of both PSA and DRE. Decomposing the total cost by diagnosis, treatment, and complications reveals that, in both strategies, medical direct costs of treatment constitute the largest share of total costs.

### Cost of prostate cancer screening by different age groups

As the total and average costs were lower in the PSA and DRE screening strategy, the calculation of PCa screening costs was conducted separately for each age group based solely on this approach. According to Table 4, the highest total screening costs were observed in men aged 40–49. Additionally, screening for men aged 40–69 had the lowest average cost, while the highest average cost was observed in men aged 80 and above.

**Table 4:** Total cost of prostate cancer screening with PSA & DRE by age group (USD)

Subject	Amount ($)				
	40–49 Age	50–54 Age	55–69 Age	70–79 Age	+ 80 Age
**Screening Medical Cost**					
PSA Test	14,164,896	6,129,034	8,382,255	3,520,149	1,377,578
Biopsy & complication	404,028	856,840	1,043,621	998,729	557,211
Treatment & complication	554,652	3,069,415	3,599,235	3,493,452	1,945,061
Total Direct Medical Cost	15,123,576	10,055,288	13,025,111	8,012,330	3,879,851
**Screening non-Medical Cost**					
Direct Non-Medical Treatment Costs	4,238,705	2,616,244	3,591,199	2,079,671	983,471
Direct Non-Medical Diagnosis Costs	3,984,791	1,605,791	2,360,705	902,049	326,487
Total Direct Non-Medical Costs	8,223,497	4,222,035	5,951,904	2,981,720	1,309,957
**Total Costs**	23,347,073	14,277,323	18,977,014	10,994,050	5,189,808
Average Cost of Screening Per man	5	8	7	11	14

### Men’s willingness to pay by different age groups

The men’s willingness to pay across age groups, which was extracted from our prior study, is outlined in Table 5. For more details, refer to the relevant study [27].

### Cost-benefit analysis

Based on Willingness to Pay (WTP) using both basic and complementary information, PCa screening with PSA for all men showed NCBI values of $9.14 and $7.67 per screened case, respectively. Additionally, PCa screening with PSA and DRE had NCBI values of $11.1 and $9.7 for men with basic and complementary information, respectively (Table 5).

**Table 5:** Average cost, benefit and NCBIs by age groups ($)

	All men	(40–49)	(50–54)	(55–69)	(70–79)	80+
**Benefit**						
Average WTP-Basic information	17.79	18.81	17.89	18.84	8.79	6.8
Average WTP-Complementary information	16.32	18.54	12.58	9.68	7.66	0.53
**Cost**						
Average Cost- Screening by PSA	8.65	8.19	13.16	12	22.63	32.41
Average Cost- Screening by PSA & DRE	6.6	5	7.7	6.9	10.6	14
**Nest benefit- screening by PSA**						
Net cost-benefit index-basic information	9.14	10.62	4.86	6.84	-13.84	-25.61
Net cost-benefit index- Complementary information	7.67	10.35	-0.58	-2.32	-14.97	-31.88
**Net benefit-screening by PSA & DRE**						
Net cost-benefit index-basic information	11.1	13.8	10.19	11.9	-1.81	-7.2
Net cost-benefit index- Complementary information	9.7	13.5	4.9	2.78	-2.94	-13.47

The NCBI starts positively at $9.14 for basic information in the case of PSA screening. It decreases to $7.67 for complementary information, but remains positive, signifying a favorable balance between screening cost with PSA and the benefit measured by willingness to pay. On the other hand, for screening through PSA and DRE, the NCBI increases to $11.1 with basic information and $9.7 with complementary information. This clear trend indicates that screening through PSA and DRE emerges as the dominant strategy for prostate cancer (PCa) screening.

The NCBI for the dominant strategy with basic information is positive, indicating a favorable balance between the screening cost with PSA and DRE and the benefit measured by willingness to pay. However, the inclusion of complementary information results in a decrease in the NCBI for all men and all age groups.

The NCBI remains positive for individuals aged 40–69, suggesting a benefit outweighing the cost. The highest NCBI is observed in the youngest age group (40–49), reaching $13.8 for basic information and $13.5 for complementary information. This discrepancy indicates that basic information contributes to a more favorable balance in this age group. In contrast, the lowest Net Cost-Benefit Index is observed in individuals aged 70 and above, showing a negative value and indicating a less favorable balance. The highest net negative benefit was observed in the 80 + age group.

This result illustrates a consistent decreasing trend for both basic and complementary information as age increases.

### Sensitivity analysis

**Fig. 2:**
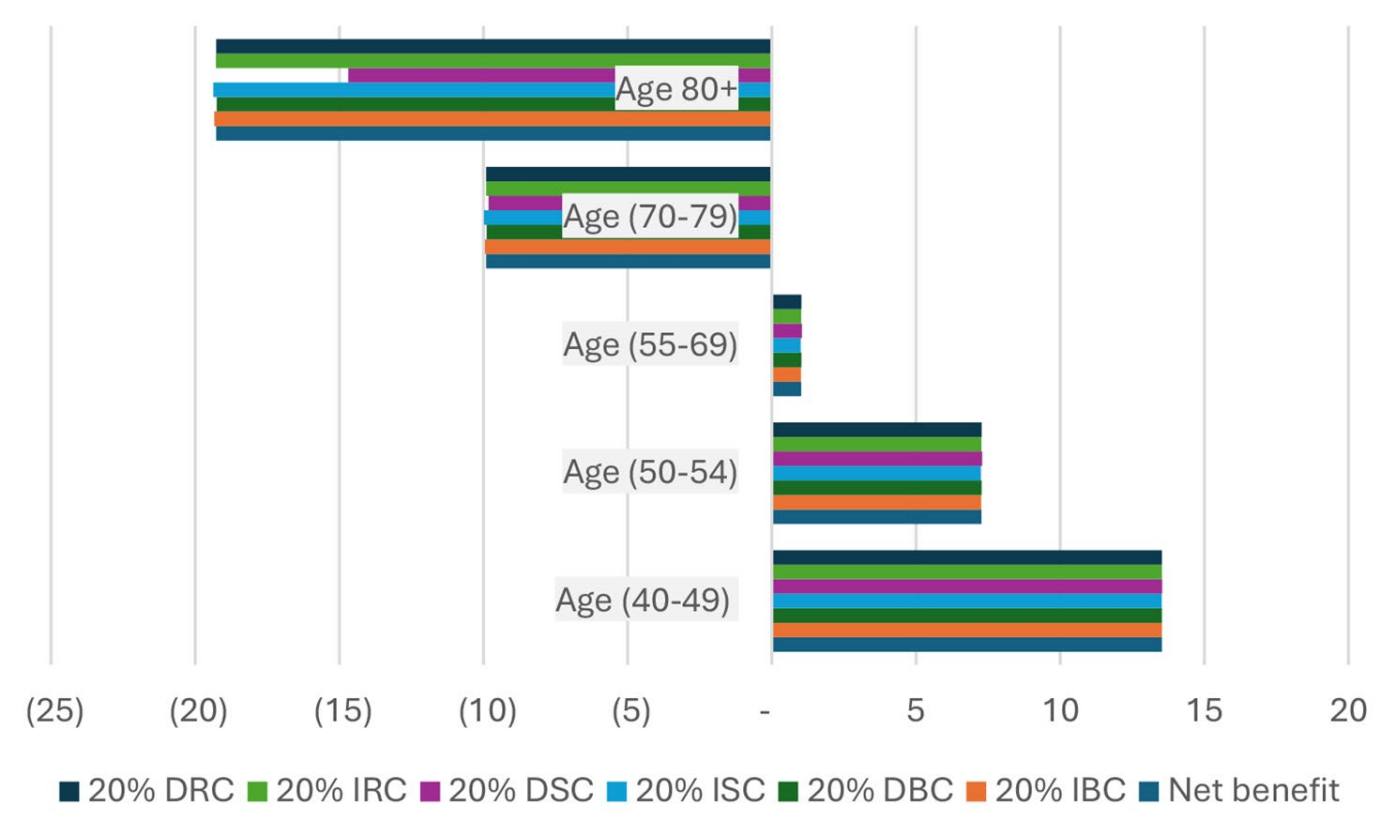
Sensitivity analysis for NCBI of men PCa screening by PSA and DRE: in case of Basic Information. **DRC**: Decrease Radiotherapy Complication, **IRC**: Increase Radiotherapy Complication, **DSC**: Decrease Surgery Complication, **ISC**: Increase Surgery Complication, **DBC**: Decrease Biopsy Complication, **IBC**: Increase Biopsy Complication

**Fig. 3:**
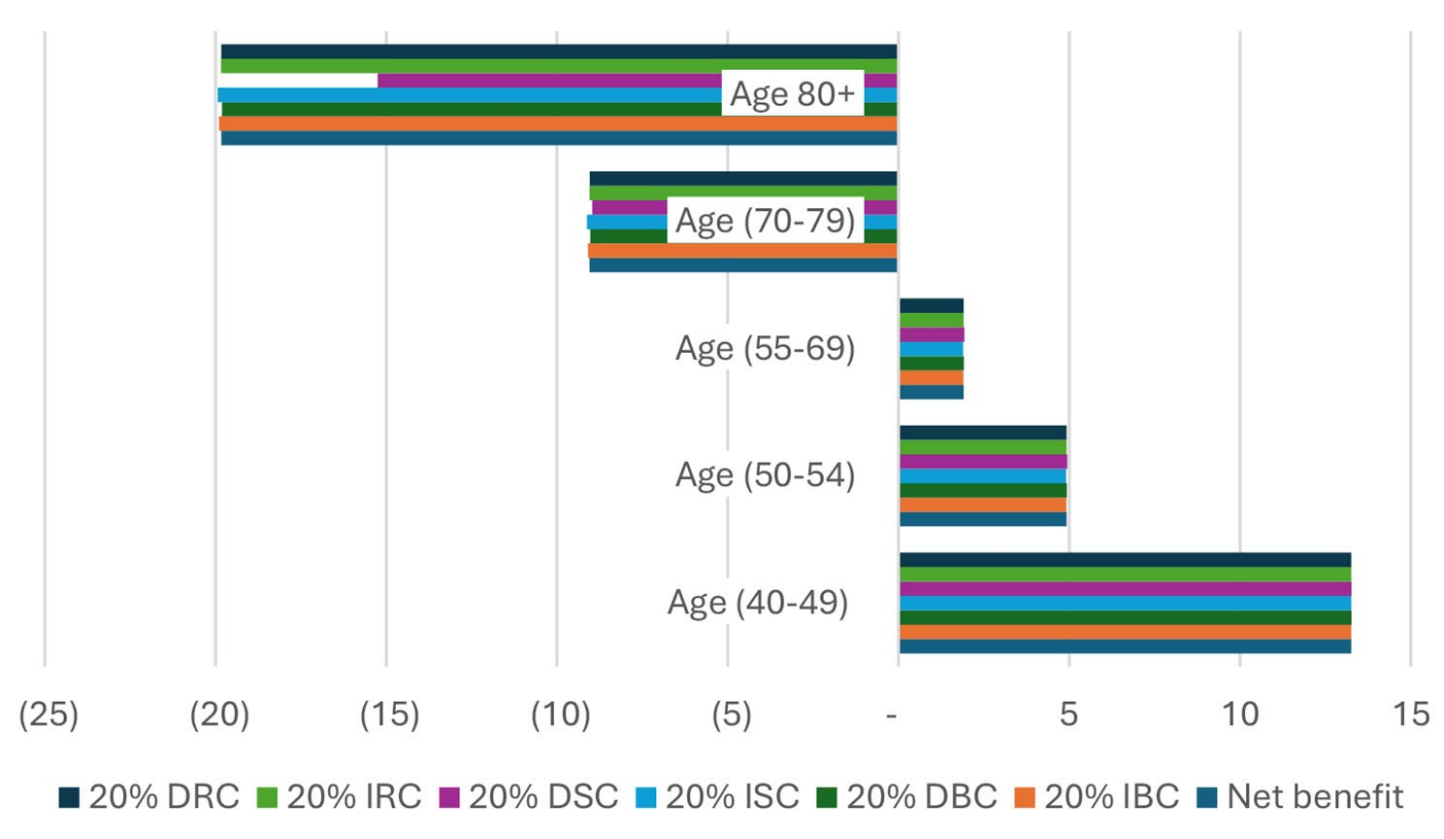
Sensitivity analysis for NCBI of men PCa screening by PSA and DRE: in case of Complementary Information. **DRC**: Decrease Radiotherapy Complication, **IRC**: Increase Radiotherapy Complication, **DSC**: Decrease Surgery Complication, **ISC**: Increase Surgery Complication, **DBC**: Decrease Biopsy Complication, **IBC**: Increase Biopsy Complication

Sensitivity analysis was conducted focusing on the combined PSA and DRE screening strategy, as illustrated in Figs. 2 and 3 below.

The results of the sensitivity analysis revealed that the NCBI for each prostate cancer (PCa) screening did not undergo significant changes. The findings remained robust and consistent despite the variations introduced. Importantly, the sign of NCBI remained unchanged for all age groups, regardless of the availability of basic or complementary information.

In summary, the sensitivity analysis demonstrated the resilience of the results, emphasizing the stability and reliability of the NCBI across different scenarios and age groups, even when subjected to variations in the potential complications of the screening and treatment processes.

## Discussion

Our exploration into prostate cancer screening, specifically using Prostate-Specific Antigen (PSA) and Digital Rectal Examination (DRE), has unraveled diverse economic implications. In this section, we provide a concise overview of our main findings before delving into the details. Our analysis centers on the NCBI and its variations across age groups, offering valuable insights into the economic feasibility and considerations for these screening strategies.

Incorporating Digital Rectal Examination (DRE) alongside Prostate-Specific Antigen (PSA) screening has been shown to offer significant economic benefits, owing to its heightened sensitivity, specificity, and diagnostic accuracy in detecting prostate cancer [21]. Numerous studies have highlighted the enhanced accuracy achieved when PSA is combined with DRE in prostate cancer detection [28–32]. While the exact reduction in unnecessary biopsies may vary - reported to be 12% in the study by Littrup [33] and even higher in our own research - our analysis emphasizes the tangible economic advantages derived from integrating DRE into the screening process. These findings are in line with recommendations put forth by the World Health Organization (WHO) [30], which advocate the joint utilization of PSA and DRE as a prudent and economically viable approach. Similarly, several studies [34–36] have lent support to the effectiveness of combining PSA with DRE for improved prostate cancer detection, a conclusion further bolstered by our own investigation.

Notably, the NCBI screening was positive for all men, indicating economic justifiability but their result was different for different age groups.

Despite accounting for different PSA threshold levels in various age groups, adding DRE to screening leads to notable variations in total and average costs and the NCBI across age categories in both screening scenarios. The results underscore the impact of age group composition on the economic outcomes of prostate cancer screening, aligning with the findings in literature [26]. 

The study gauged the benefits of early detection through Willingness to Pay (WTP), revealing that, in screening with DRE and PSA tests, the population aged 40–69 experienced a net positive benefit when provided complementary information, particularly in the age range of 40–54 it is high. This can be attributed to the reduction in WTP variance, and the cost-effectiveness achieved by combining DRE with PSA testing. In comparison to the relevant study by Rao [20], our approach, which takes into account false positive and negative results in willingness-to-pay (WTP) assessments, enhances the reliability of the findings.

Screening for men aged 70 and above was consistently deemed economically unjustified. The lowest NCBIs were observed in the 70–79 and 80 + age groups, likely influenced by reduced WTP due to limited income post-retirement, shorter life expectancy, higher probabilities of comorbidities, and increased screening costs. The findings are consistent with the existing literature, which recommends that screening beyond a certain age should be stopped to overcome overdiagnosis and associated high costs [26, 37].

Conversely, the age groups of 40–49 and 50–54 exhibited the highest NCBI, emphasizing the economic feasibility of screening in these cohorts, especially in a situation without financial limitations. However, it’s essential to note that despite the positive NCBI in the 40–49 age group, caution is warranted in situations with financial limitations. Various studies and guidelines, such as those by the European Urological Association (EAU), recommend initiating screening at age 50 [38]. The recommendation by other aligns with the consensus that initiating prostate cancer screening at the age of 50 and older is optimal, with no additional benefits observed for men commencing screening at age 40 instead of 50 [39]. The apparent contradiction with previous studies concerning this age group may stem from two primary aspects. Firstly, our study measured benefits based on stated Willingness to Pay (WTP), while prior studies utilized alternative criteria [20, 40, 41], just one study [18] found to have used WTP for measuring benefit just for PSA test as screening intervention. Secondly, previous studies aimed to enhance the probability of early detection, while our approach gauged the value of early detection from the perspective of men.

The sensitivity analysis results bolster the credibility of our study, affirming the consistent economic justification of PSA and DRE screening across diverse age groups. Our sensitivity analysis findings align with previous research [42], which evaluated a range of probabilities for potential complications, further reinforcing our study’s alignment with existing literature.

The study suggests that policymakers should carefully assess the impact of increased information levels on the target population’s willingness to participate in screening. It highlights that additional information may not necessarily lead to a significant improvement in screening acceptance and attendance [27], questioning the need for substantial financial resources to enhance public awareness initiatives.

### Limitations

- It was not possible to interview patients in extracting direct non-medical costs during the treatment phase. As the data was extracted from the SEPAS system of the Ministry of Health and due to ethical considerations, patients’ personal information such as their job, address and telephone number were not available to the research team. Therefore, this information was extracted from other studies conducted for Iran.
- The costs of complications consist of cases that occur shortly after treatment and do not include the cost of subsequent visits.
- Due to the very low probability of biopsy complications, no bills were found in the SEPAS system to the relevant action code. The unit cost of these complications was calculated based on the government tariff and was considered the same for the studied sample.

## Conclusion

In conclusion, this study strongly advocates the implementation of prostate cancer screening through the combined use of PSA and DRE. The findings suggest that, without financial limitations, screening for men between the ages of 40–69 is economically justified. However, when considering financial resource limitations, it is advisable to prioritize screening based on suggested age categories. Specifically, various studies and guidelines recommend initiating screening at age 50. Following these recommendations, the suggested age priorities for screening based on our results are as follows: men aged 50–54 as the primary priority, followed by men aged 55–69 as the secondary priority, and men aged 40–49 as the tertiary priority. These recommendations carry vital policy implications, suggesting that allocating public resources to screening men aged 69 and above (and those under 40) may not be economically justified. Moreover, the results provide a rationale for health insurers to consider covering the cost of prostate cancer screening, offsetting treatment costs, and justifying the investment. Considering Willingness to Pay (WTP) as the basis for benefit calculation, it becomes evident that the first to third age priorities present the highest potential for out-of-pocket payments for screening. In the absence of sufficient resources from health insurers and government budgets, individuals within these age groups may be more inclined to bear the screening costs, providing an opportunity for the government to complement the screening program. While this study was conducted statically over a specific period, its results can also be analyzed in a dynamic context. The initiation and continuation of the screening program may foster a preventive habit among current 40-year-old men, who would undergo screening 29 times annually until reaching the age of 69. This habitual engagement, coupled with ongoing justification programs, may lead to a sustained desire for screening even after the age of 69. In such a scenario, out-of-pocket payments might not hinder continued screening unless individuals are dealing with other chronic conditions or do not foresee a longer life for any reason.

The present study recommends policymakers evaluate the impact of increased information on participation in screening. It suggests that more information might not significantly enhance attendance, raising doubts about the necessity for substantial financial resources in public awareness initiatives.

## Data Availability

Data is provided within the manuscript or supplementary information files.

## Abbreviations

NCBIs: Net Cost-Benefit Index
PCa: Prostate Cancer
PSA: Prostate Specific Antigen
DRE: Digital Rectal Examination
WTP: Willingness to Pay
CVM: Contingent Valuation Method
